# Probability mapping of scarred myocardium using texture and intensity features in CMR images

**DOI:** 10.1186/1475-925X-12-91

**Published:** 2013-09-22

**Authors:** Lasya Priya Kotu, Kjersti Engan, Karl Skretting, Frode Måløy, Stein Ørn, Leik Woie, Trygve Eftestøl

**Affiliations:** 1 Department of Electrical Eng. and Computer Science, University of Stavanger, Stavanger 4036, Norway; 2 Department of Cardiology, Stavanger University Hospital, Stavanger 4011, Norway

## Abstract

**Background:**

The myocardium exhibits heterogeneous nature due to scarring after Myocardial Infarction (MI). In Cardiac Magnetic Resonance (CMR) imaging, Late Gadolinium (LG) contrast agent enhances the intensity of scarred area in the myocardium.

**Methods:**

In this paper, we propose a probability mapping technique using Texture and Intensity features to describe heterogeneous nature of the scarred myocardium in Cardiac Magnetic Resonance (CMR) images after Myocardial Infarction (MI). Scarred tissue and non-scarred tissue are represented with high and low probabilities, respectively. Intermediate values possibly indicate areas where the scarred and healthy tissues are interwoven. The probability map of scarred myocardium is calculated by using a probability function based on Bayes rule. Any set of features can be used in the probability function.

**Results:**

In the present study, we demonstrate the use of two different types of features. One is based on the mean intensity of pixel and the other on underlying texture information of the scarred and non-scarred myocardium. Examples of probability maps computed using the mean intensity of pixel and the underlying texture information are presented. We hypothesize that the probability mapping of myocardium offers alternate visualization, possibly showing the details with physiological significance difficult to detect visually in the original CMR image.

**Conclusion:**

The probability mapping obtained from the two features provides a way to define different cardiac segments which offer a way to identify areas in the myocardium of diagnostic importance (like core and border areas in scarred myocardium).

## Introduction

**Figure 1:**
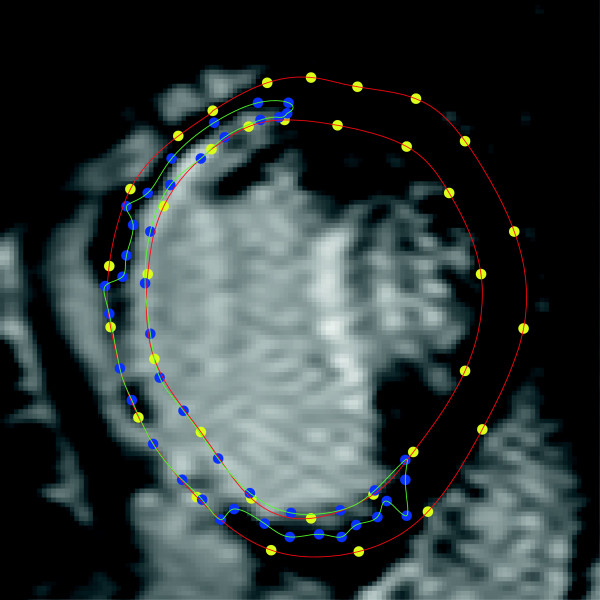
**Cropped short-axis CMR image showing manual segmentation of the myocardium and scar tissues. **The yellow and blue dots in the image are manually marked (by Cardiologist) coordinates to segment myocardium and scar. The red and green contours generated by cubic spline interpolations of the above coordinates show myocardium and scar tissues respectively.

Patients who have suffered but survived myocardial infarction (MI) may subsequently suffer a possibly disabling or fatal cardiac arrhythmia. Late Gadolinium (LG) enhanced Cardiac Magnetic Resonance imaging (CMR) is used for assessing morphology of the myocardium in patients after MI. An example image is depicted in Figure 1 with myocardium and scar delineated by green and red lines, respectively. The myocardial muscle fibers are completely dead at the core area of the scar. Thus, the core area does not react to the electrical signals propagating through the heart muscle telling the heart to contract.

At the border areas, however, the muscle fibers are not all dead. The electrical signals will be disturbed in these areas possibly causing reentries and sometimes arrhythmias. The latter gives us reason to believe that the border areas are a very important part of the scar, and thus good ways of defining and visualizing border areas would be beneficial in many situations. Previous works [1,2] emphasize the importance of the border area (also referred to as peri-infarct area, or gray zone area) being a major determinant for identifying patients at risk of arrhythmia. The heterogeneous nature of cardiac segments is related to various diagnoses and prognosis. For example, Risk of serious arrhythmias and better indication for implantation of implantable cardioverter defibrillator (ICD) [3], heart rate of ventricular tachycardia [4], risk and degree of heart failure, indication of implantation of pacemakers in order to synchronize cardiac contractions [5] etc. Our main focus in this work is to develop methods to visualize, define and analyze the myocardium to identify regions of diagnostic importance in the myocardium.

### Earlier reported work

To our knowledge, visualization of the heterogeneous nature of the myocardium is mostly based on thresholding methods described in the literature where thresholds are defined at intensity levels corresponding to some percentage of the maximum intensity level inside the scar area [1-3]. Recently, Shokrollahi *et al.* was able to divide the scarred myocardium of animals into core and gray zones using fuzzy clustering algorithm and validated the results using in-vivo electro-anatomical voltage mapping [6]. Some previous publications reported automatic segmentation of scarred myocardium in late enhanced magnetic resonance images [7-9]. The segmentation of scar itself is an important application as the scar size contains information useful for finding the patients with high and low risk of arrhythmia [3]. Also, scar size is largely responsible in ventricular remodeling [10]. Dicki *et al.* use support vector machine (SVM) to classify the scar and the myocardium in delayed enhancement magnetic resonance images in [7] using three features. The three features used in [7] depend on the intensity of scar, myocardium and the whole CMR image.

From our group’s previous work [11], it is shown that patients with high and low risk of getting arrhythmia can be classified using textural measures from gray-level co-occurrence matrices. In our recent work [12], we presented some preliminary results where dictionary learning and sparse representations were used for representing the texture in the myocardial segments followed by a classification.

### The proposed probability mapping

All the above discussed previous work have sought to identify methods to segment the hyper-enhanced area of the myocardium. However, by only delineating the hyper-enhanced myocardium, important information concerning the tissue within the infarct territory is not provided. Hence, our focus is on developing a method that provides probability values as a means to distinguish the areas of the myocardium rather than the crisp definitions offered by a scar vs no scar segmentation.

In this work, we propose a technique for transforming the scarred myocardium in the CMR image to a probability map to indicate varying degrees of its functioning capabilities: where high and low probabilities will indicate the scarred tissue and normal tissue, respectively. Section ‘Calculation of probability maps using Bayes rule’ describes the general principles for calculation of the probability maps, where each pixel is the posterior probability of that pixel being scar compared to healthy myocardium. The posterior probabilities are calculated using Bayes rule with the help of features from the myocardium. Features are calculated pixel by pixel based on a local neighborhood and is explained in Section ‘Extraction of features’. We have based our experiments on two features; a local mean (direct current, DC) that will correspond well to how the doctors manually segment the scar, and a textural feature using sparse representations, that might reveal information regarding gray zone areas.

Section ‘Experiments and results’ shows the experimental framework and examples of probability maps for both features. Also in section ‘Experiments and results’, cardiac segments example is demonstrated to show the diagnostic importance of texture feature probability maps. Finally, the paper is concluded in section ‘Conclusion’.

## Calculation of probability maps using Bayes rule

The class specific probability density functions (PDFs) modeling specific feature vector values known to be located within scar or healthy myocardial areas are estimated as *p*(*v*|*scar*) and *p*(*v*|*myo*), respectively. According to Bayes rule [13], the probability of a pixel being in the scar area is related to the density function and the prior probability function:

(1) $$P(\text{scar}|v)\;=\;\frac{\left(P(\text{scar})p(v|\text{scar})\right)}{p(v)},$$

where *p*(*v*) = *P*(*scar*)*p*(*v*|*scar*)+*P*(*myo*)*p*(*v*|*myo*), and *v* is a feature vector. The prior of scar, *P*(*scar*), reflects the mean value of the scar size in the training data set, and *P*(*scar*) is calculated as the ratio of the number of pixels present in the scar with respect to the myocardium in the entire training data.

The parameters in the class specific PDFs can be estimated using parametric or non-parametric methods, and here they are calculated using maximum likelihood (ML) estimation [14] which is a method of low complexity.

Our primary focus is deriving the probability mapping, and there is a strong link to segmentation as we define the functions so that they should be optimal in the sense of discriminatively expressing the properties of the scar and normal tissue. The minimum error classifier corresponds to classifying as scar when *P*(*scar*|*v*)>*P*(*myo*|*v*). Therefore, we evaluate the features and feature combinations used in various mapping with respect to their segmentation capability. As we will discuss later, this might not be entirely fair as in our case; features reflecting the cardiologist’s crisp decisions are favored.

**Figure 2:**
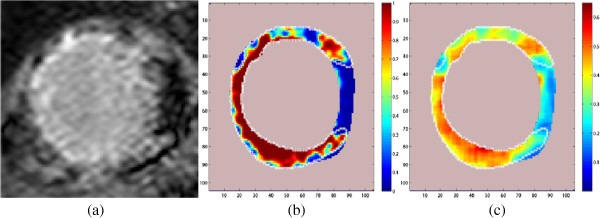
**Probability maps of the scarred myocardium in CMR images of a patient implanted with ICD using the DC and texture features. (a)** The cropped CMR image containing left ventricle. **(b)** Probability map using the DC value as feature: Dark Red 100%–90%, red 90%–80%, yellow 70%–60%, green 60%–50%, cyan 50%–30% and blue 20%–0% probability of being scar. The other image parts are shown in light pink color. **(c)** Probability map using the texture feature. The same color code described above is used here. White contour shows the cardiologists segmentation of scar.

The extraction of feature vectors for myocardium is illustrated in section ‘Extraction of features’. Feature vector *v* of the myocardium in general can be represented as:

(2) $$v\;=\;[{\text{feature}}_{1},\;{\text{feature}}_{2}\;\ldots \;{\text{feature}}_{d}],$$

where *d* is the dimension of the feature vector *v*. Finally, the probability maps are obtained by regenerating a new image such that each pixel in the myocardium is given by the value of *P*(*scar*|*v*) as calculated for that pixel. The probability values range from 0–1 and they are displayed using a color code shown in Figure 2(b) and (c). The shades of red and yellow in the color code represents high probability of scar and shades of green and blue represents low probability.

### Training and testing

**Figure 3:**
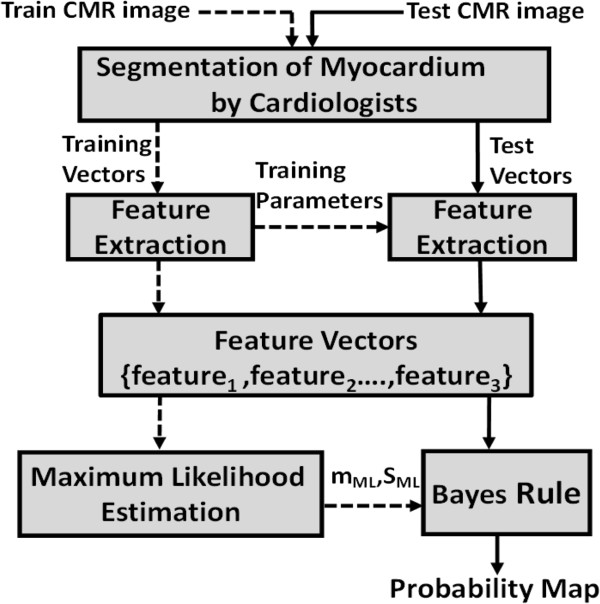
Training (dashed lines) and test (solid lines) phases involved in computing the probability maps of the scarred myocardium.

The process of generating probability maps is depicted in Figure 3. The whole CMR data set is divided into training and testing images. In the training process, training features are extracted along with some training parameters required in the testing phase. In the next step, the calculated training feature vectors are used for estimating the PDFs of scar and myocardium region using ML. The training process and its outputs are indicated by the dashed lines. The probability maps are generated on test images, different from the training images, and this process is indicated by solid lines. The extraction of features for training and testing phases are explained in section ‘Extraction of features’.

## Extraction of features

As discussed in section ‘Introduction’, two features, DC value and texture feature are used to generate probability maps of the scarred myocardium in this work. The detailed process of extracting the two features are discussed in the following sections ‘DC Feature’ and ‘Dictionary-based textural feature’.

### DC Feature

Historically, in electronics field, the *mean* is commonly referred to as DC (direct current) value [15]. Because of the intensity differences between the healthy and scarred myocardium tissues in the LG enhanced CMR images, DC value *dc*(*i*,*j*) is one of the features explored for developing the probability maps. This is probably the feature that would mimic the cardiologists segmentation best since they use the intensity values of scarred myocardium in CMR image to segment the scarred areas manually. We define the feature $\text{dc}(i,j)=\text{mean}(I_{\sqrt{N}\times \sqrt{N}}(i,j))$ (where $I_{\sqrt{N}\times \sqrt{N}}$ is the neighborhood around the pixel *I*(*i*,*j*)), such that a new image *I*_*dc*_, with *dc*(*i*,*j*) as values at pixel position (*i*,*j*) is made with a sliding window averaging over the image.

### Dictionary-based textural feature

A dictionary *D*, is an ensemble of a finite number of atoms, *d*^(*k*)^, and can be used to represent signals. A linear combination of some of the atoms in the dictionary gives exact or approximate representation of the original signal. Let a column vector *x* of finite length *N* represent the original signal. The dictionary atoms are arranged as columns in an *N* × *K* matrix *D* where *K* > *N*. The representation or the approximation of the signal, $\tilde{x}$ and the representation error, *r* can be expressed as:

(3) $$\tilde{x}=\overset{K}{\underset{k=1}{\sum }}w(k)d^{\left(k\right)}=\text{Dw},\;r=x-\tilde{x}=x-\text{Dw},$$

where *w* is sparse coefficient vector. In a sparse representation, only a small number of the coefficients *w*(*k*) are allowed to be nonzero. Finding the sparse coefficient vector can be a NP-hard (Non-deterministic Polynomial-time hard) problem and formulated as:

(4) $$w_{\text{opt}}=\underset{w}{\text{argmin}}∥x-\text{Dw}{∥}_{2}\;\text{s.t}\;∥w{∥}_{0}\leq s.$$

The pseudo-norm ∥.∥_0_ is the number of nonzero elements and, *s* denotes the sparseness. A vector selection algorithm called Order Recursive Matching Pursuit (ORMP) algorithm is used for sparse approximation [16].

Dictionary learning is the task of learning or training a dictionary on an available training set such that it adapts well to represent that specific class of signals. The training vectors, *L* and the sparse coefficients are arranged as columns in the matrices *X* and *W*, respectively. The objective in dictionary learning is to give a sparse representation of the training set *X*in order to minimize the sum of the squared error. The cost function is formulated as:

(5) $$\underset{D,W}{\text{argmin}}\;F(D,W)=\underset{D,W}{\text{argmin}}\underset{i}{\sum }∥r_{i}{∥}_{2}^{2}\;\text{s.t}\;∥w{∥}_{0}\leq s.$$

This is a very hard optimization problem. Recursive Least Squares Dictionary Learning Algorithm (RLS-DLA), [17] is used for dictionary learning in all our experiments. A brief explanation of the algorithm is given in Appendix.

Sparse representations and learned dictionaries have been shown to work well for texture classification by Skretting and Husøy in [18] and by Mairal *et al.*[19]. Texture in a small image patch can be modeled as sparse linear combination of dictionary atoms in Frame Texture Classification Method (FTCM) presented by Skretting *et al.* in [18]. FTCM is developed by modeling a texture as a tiled floor where all the tiles are identical. The color or the gray level, at a given position in the floor is given by an underlying continuous periodic two-dimensional function. It is shown based on this model that a vector from spatial neighborhood is indeed a sparse combination of finite dictionary atoms.

The algorithm for sparse representation in our work proceeds as follows in the training phase: Consider the myocardium in a CMR image, *I* that contains two texture classes: healthy and scarred myocardium. The training vector, *y*_*l*_ of length *N* is made from that specific pixel and its neighborhood, $\sqrt{N}\times \sqrt{N}$ for each pixel in the training image. In the training set, each pixel is classified into a specified texture class. Then, the dictionaries, *D*_*s*_ and *D*_*m*_ are trained for the predefined texture classes (scar and myocardium) using RLS-DLA. Every training vector, *y*_*l*_ is then represented sparsely using ORMP vector selection algorithm [16] twice, both with *D*_*s*_ and *D*_*m*_. The residuals (or representation errors), *R*_*s*_ and *R*_*m*_, are calculated for each pixel in the myocardium of a training image as:

(6) $$R_{s}(i,j)=∥y_{l}-D_{s}w_{l}^{s}∥\;\text{and}\;R_{m}(i,j)=∥y_{l}-D_{m}w_{l}^{m}∥,$$

where $w_{l}^{s}$ and$w_{l}^{m}$ are sparse coefficient vectors.

The residuals, *R*_*s*_ and *R*_*m*_, are combined to form the texture feature used in this work. The texture feature is calculated as the ratio of the residual of *D*_*m*_ to the sum of the residuals of *D*_*s*_ and *D*_*m*_:

(7) $$R_{p}(i,j)=R_{m}(i,j)/(R_{s}(i,j)+R_{m}(i,j)).$$

Pixel by pixel *R*_*p*_ value can be interpreted as the scaling of residuals *R*_*s*_ and *R*_*m*_. Smaller values of *R*_*p*_ means that the pixel is not likely to be scar (i.e. healthy myocardium), and larger values means that it is likely to be scar or border area. The texture feature, *R*_*p*_(*i*,*j*) from the training set is used to estimate the PDFs for the probability maps computation. In the testing phase, texture feature vectors are collected from the test image set, in the same way as training feature vectors. In Figure 3, training and classification parameters are indicated. In fact, these training parameters are the dictionaries, *D*_*s*_ and *D*_*m*_. Using *D*_*s*_ and *D*_*m*_ learned in the training phase, the residual images, *R*_*s*_ and *R*_*m*_ are calculated for test images. Texture is not pixel-by-pixel local except in the edge between two textures; thus some sort of smoothing is used on the test residual images before calculating the probability maps [18]. The test residual images *R*_*s*_, and *R*_*m*_ are smoothed using an *a* × *a* pixels separable Gaussian low pass filter with variance *σ*^2^ before calculating *R*_*p*_.

## Experiments and results

The CMR images used in our experiment were provided by the Department of Cardiology in Stavanger University Hospital and were obtained from 44 patients with myocardial infarction. 24 patients had old MI and ICD was implanted in all patients as primary- or secondary prophylaxis. This group was defined as the high risk arrhythmic group. The remaining 20 patients had MI one year prior to CMR imaging and during this observation period, no reported incidents of serious arrhythmias occurred. This group was defined as the low risk arrhythmic group. The study was approved by the Regional Ethics Committee and informed consent was obtained from all patients.

All the CMR images were obtained from 1.5 Tesla Philips Intera machine. Images were acquired with a pixel size of 0.82 × 0.82 *mm*^2^, covering the whole ventricle with short-axis slices of 10 *m* thickness, without inter-slice gap. The LG enhanced CMR images were stored according to the digital imaging and communications in medicine (DICOM) format with 512 × 512 pixel resolution. The number of image slices with visible scar in each patient varies depending on the size of the scar and the scar size varies from one slice to the other. Only short-axis image slices were used in our experiments. Out of the total 444 CMR images belonging to the 44 patients, 11 CMR images of poor quality due to motion and artifacts were not included. An experienced cardiologist delineated the borders of the myocardium and the infarction tissues as shown in Figure 1. The resulting set of segmented images were further split into labeled training and test sets. All our experiments were carried out in MATLAB. The CMR training and test images were cropped from 512×512 original CMR image so that they contain only left ventricle with myocardium and infarction areas to save execution time. Preprocessing of any kind was not used on these cropped images.

In all the CMR images, we took into account only the myocardium segmented by the cardiologists. Patients with high risk of getting arrhythmia have profound scars with possibly large gray-zone areas. We therefore anticipated that images from these patients contained the information we wanted to represent in the dictionaries: information from the myocardium, the core area of scars, and the gray-zone areas. Hence, the high risk patients group was used in the training phase. From the group of 24 high risk patients, 6 patients were randomly selected to form a training set, whereas the remaining 18 high risk patients were used to form a test set. Two sets of training vectors were generated, one from the scar, and one from the healthy myocardium.

This section is organized as follows: Sections ‘Probability maps obtained using DC feature, *dc*(*i*,*j*)’ and ‘Probability maps obtained using texture feature, *R*_*p*_(*i*,*j*)’ explains the processes of calculating the probability maps using DC and texture feature respectively. Section ‘Examples of probability maps obtained from DC and texture feature’ discusses the examples of probability maps obtained in our experiments. Section ‘A possible application - cardiac segments’ illustrates how probability maps obtained with texture feature can be used to define cardiac segments. Section ‘The discriminatory power of the DC and texture features’ considers the discriminative power of the DC and texture features.

### Probability maps obtained using DC feature, *dc*(*i*,*j*)

The neighborhood size 3×3 was used to form training vectors as explained in ‘DC Feature’. The same neighborhood size must be used while training and finding the DC images *I*_*dc*_. The DC images obtained from the training images were used to form the training feature set. The parameters of the class specific PDFs: the mean and the standard deviation were found using ML estimation using the training feature set. DC-values were scaled to have zero mean and unit variance before finding the ML estimates. The scaling coefficients from the training were stored to scale the test vectors. The probability maps of the scarred myocardium were calculated using Bayes rule as explained in section ‘Calculation of probability maps using Bayes rule’. An example of a probability map obtained using the DC feature with color code is shown in Figure 2(b). Note the sharp transition from the segmented scar to the myocardium.

### Probability maps obtained using texture feature, *R*_*p*_(*i*,*j*)

**Figure 4:**
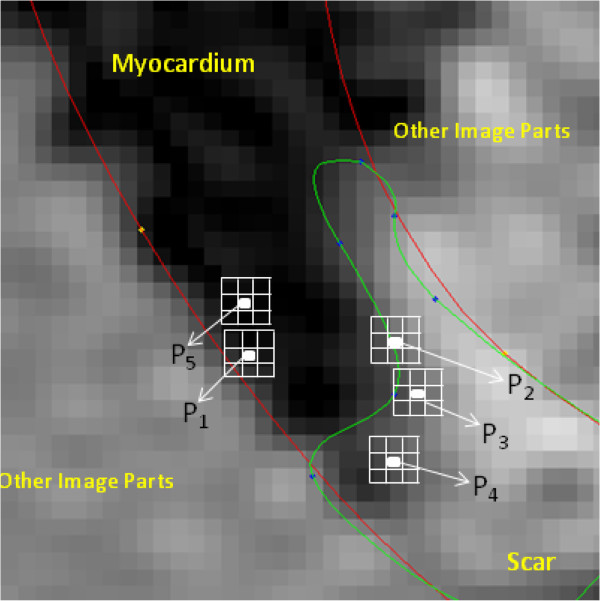
**Training vectors are generated from each pixel as long as that entire neighborhood lies within one texture region.** The neighborhoods of pixels *P*_1_, *P*_2_ and *P*_3_ include more than one texture, and the corresponding feature vectors are excluded from the training set. *P*_4_ and *P*_5_ have the entire neighborhood within one texture, and hence the corresponding feature vector is included in that texture’s training set.

The training vector and test vectors were generated in the same way as in the DC feature experiment using the same neighborhood size 3×3. Consider the pixels on the border zones, their neighborhood extends into other regions that are not under consideration. If we use training vectors from border regions, the dictionaries might learn the texture properties of other regions along with the texture properties they are intended to learn. So, the training vectors for the pixels whose neighborhood span other regions were not considered in our experiments. This is depicted in Figure 4.

The dictionaries were learned on the training vectors generated from scar and healthy myocardium using RLS-DLA. The dictionary size of 9 × 90 atoms was used in our experiments. The initial dictionaries were formed by randomly selecting 90 vectors of length 9 from the training sets. Dictionary size was selected based on previous experience, and it should be *K* > *N* < < *L*. The forgetting factor is initialized to 0.995 and slowly increased towards 1 according to the exponential method described in [17]. If the number of dictionary atoms used to represent the image patch increases, then the residual decreases, and a large number of atoms will provide a good approximation with *any* full rank dictionary. Therefore, higher sparsity lowers the difference between the residuals of healthy myocardium and scar which in turn decreases the differentiation between the classes. The sparsity *s* used in our work is two, i.e. we used two vectors from the dictionary to represent the original image patch.

After learning the dictionaries, they were used to obtain the residual images. For each pixel in the myocardium of training images, the scaled residual *R*_*p*_(*i*,*j*) was found from residual images as explained in section ‘Dictionary-based textural feature’. The training feature set was then generated from these scaled residual values. The parameters of the PDFs to be used with the classifier was estimated from the labeled training vector set using ML estimation. The test vector set in this experiment was calculated after smoothing (using low pass Gaussian filter with *σ* = 5 and 9 × 9 window size) the test residual images. Using the ML estimates and Bayes rule, the probability maps for texture features of the scarred myocardium were obtained. An example of such a probability map with color code is shown in Figure 2(c). The color code used is explained under Figure 2. Here, the dictionaries were learned without the removal of the DC value in each image patch.

**Figure 5:**
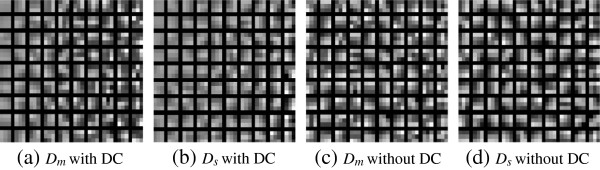
**Dictionaries of the healthy myocardium and scar: *D***_***m***_** and *D***_***s ***_**with DC value and removing DC value of 3 ×3 patches.** Absolute mean of the first derivative of the dictionary atoms: **a)***D*_*m*_ with DC- 0.3972 **b)***D*_*s*_ with DC- 0.1455 **c)***D*_*m*_ without DC- 0.7314 **d)***D*_*s*_ without DC- 0.7336.

Dictionary learning was performed with and without the removal of the DC factor from the image patch of the training set. In order to illustrate some of the texture captured by the dictionary atoms, they were plotted as image patches in Figure 5. The difference between the scar and the healthy myocardium dictionaries was visible when the DC value was not removed, and this is according to the known intensity difference between scar and healthy myocardium. To investigate the variation in the dictionary atoms used as image patches, we had calculated a measure from the first derivative of dictionary atoms in both vertical and horizontal directions. The absolute mean of the first derivative of the dictionary atoms was used to compare the scar and healthy myocardium dictionaries learned with and without DC value. The calculated absolute means are shown under Figure 5. The difference between the scar and healthy dictionaries without DC removal was observed clearly, both from the depicted dictionaries and the absolute means. The difference between the scar and healthy myocardium dictionaries trained by removing the DC value from image patches could not be observed visually. A larger block for DC removal prior to textural capturing is probably more appropriate, more similar to suppression of difference in illuminance conditions in computer vision.

**Figure 6:**
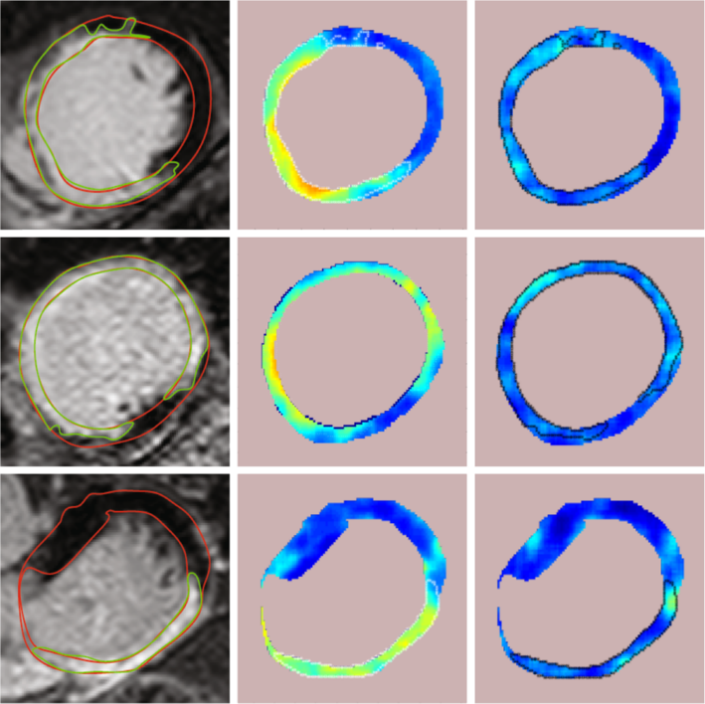
**Probability maps of the scarred myocardium in CMR images using the texture feature, *R***_***p***_**. **Column **I:** The cropped CMR images of 3 patients implanted with ICD containing left ventricle. **II:** Probability maps of the texture feature computed with dictionaries trained on image patches (used for collecting training vectors) without removing the DC value. **III:** Probability maps of the texture feature computed with dictionaries trained on image patches by removing the DC value. White and black contours shows the cardiologists segmentation of scar.

Figure 6 shows probability maps of some example CMR images and all of them were calculated using the texture feature, *R*_*p*_(*i*,*j*). In the middle column, the DC value was not removed from each image patch before training dictionaries and calculating the probabilities. In the last column, the DC value was removed from every image patch both before learning dictionaries and before finding probabilities. The DC value is obviously important, so we do not expect this experiment to provide competitive results, but we see a tendency of higher values in the scar and border areas, but not necessarily in the *core* of the scar. We expect the scar core to be very bright and could be captured easily by some sort of intensity based measure (for example the DC feature). If the physical nature of the tissue is reflected in the image texture, we can expect the scar core to be quite homogeneous. Thus, we are not surprised that the regions that are the brightest in column II were actually quite dark in column III (core), but the intermediate regions were captured fairly. We have continued with the experiments in this work by letting the dictionaries incorporate the DC value as we did not combine the DC feature, *dc* and texture feature, *R*_*p*_ into a 2D dimensional feature for computing the probability maps. Unless it is mentioned under figures of probability maps, all the probability maps of texture feature, *R*_*p*_ were computed with dictionaries learned without removing DC from every image patch.

### Examples of probability maps obtained from DC and texture feature

**Figure 7:**
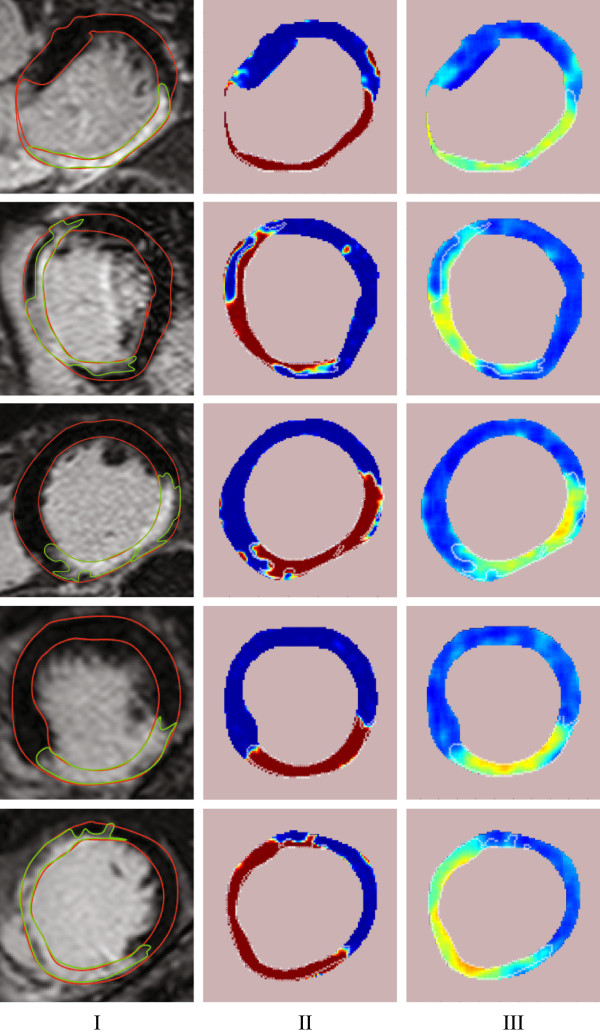
**Probability maps of the scarred myocardium in CMR images of 5 patients implanted with ICD using the DC and texture features.** Column **I:** The cropped CMR images containing left ventricle. **II:** Probability maps using DC value as the feature. **III:** Probability maps using the texture feature. Refer to the Figure 2 for the color description.

**Figure 8:**
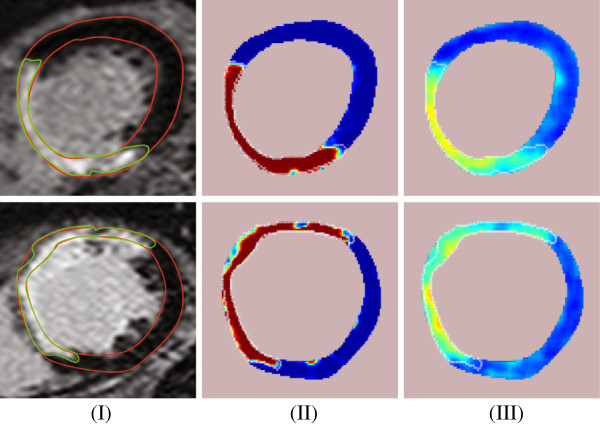
**Probability maps of the scarred myocardium in CMR images of patients without an ICD using the DC and texture features. **Column **I:** The cropped CMR image containing left ventricle. **II:** Probability map using DC value as the feature. **III:** Probability map using the texture feature. Refer to the Figure 2 for the color description.

**Figure 9:**
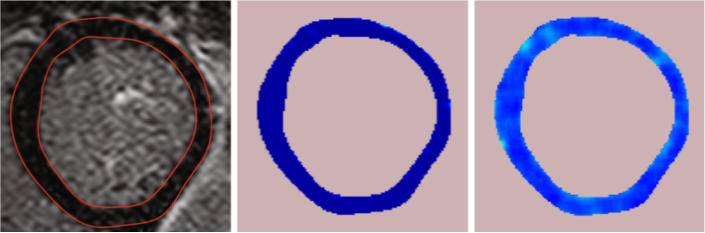
**Probability maps of the myocardium without scar in CMR images of patients with ICD using the DC and texture features. **Column **I:** The cropped CMR image containing left ventricle. **II:** Probability map of the myocardium without scar using DC value as the feature. **III:** Probability map of the myocardium without scar using the texture feature. Refer to the Figure 2 for the color description.

The second and the third column of Figure 7, shows more examples of probability maps of scarred myocardium using the DC, *dc*(*i*,*j*), and texture, *R*_*p*_(*i*,*j*), features, respectively. Figure 8 shows examples of probability maps of scarred myocardium for both DC and texture feature for the patients without ICD. This shows that the PDFs estimated for the DC and texture features from the patient group implanted with ICD were also able to produce probability maps for low risk arrhythmia patients (i.e. patients without ICD). Both the features were able to identify the healthy myocardium without scars, and an example of this is shown in Figure 9. In order to compare the probability maps obtained by the two methods, they are plotted with the same color map. Experiments of probability mapping combining the DC feature and texture feature gave almost identical probability maps as the DC feature because of the dominance of the DC feature over the texture feature. Improved ways of combining both the features will be explored in our future work.

### A possible application - cardiac segments

A cardiac segment, *CS* can be defined as a region of interest in the myocardium which might have diagnostic importance. We define a candidate cardiac segment by a lower and upper probability so that the segment consists of all pixels with probability values in the range between these. Different candidate cardiac segments might be evaluated, for example, calculating one or several features from the candidate segment and correlate result to a clinical meaningful hypothesis. We demonstrate the concept by making a comprehensive search for significant cardiac segments by comparing two patient groups and testing for statistical significance. The demonstration experiment is meant to illustrate how the probability mapping can be used to localize cardiac segments containing information discriminating between the high and low risk patient groups.

**Figure 10:**
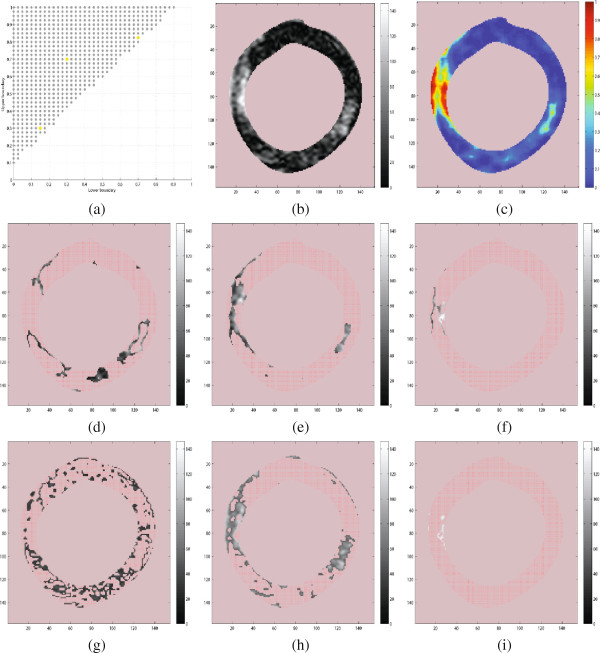
**Exploring cardiac segments of a patient implanted with ICD. (a)** The coordinates (*L*,*U*) shows all cardiac segments (CS) corresponding to the intervals *L* - *U*. **(b)** An image slice from a patient from the group with high risk of arrhythmia, **(c)** The corresponding probability map, and **(d–f)** the cardiac segments corresponding to the yellow (*L*,*U*) coordinates *CS*_1_, *CS*_2_, and *CS*_3_. Subplots **(g–i)** shows the corresponding cardiac segments derived without probability mapping.

For each patient, cardiac segments were determined by a lower boundary *L* and an upper boundary *U*. These boundaries were varied in the range 0–1 in steps of 0.025 with the restriction that *U* - *L* ≥ 0.1. Figure 10(a) illustrates this by visualizing each of the resulting candidate cardiac segment as a black dot with coordinate (*L*,*U*). For example, the three yellow dots in coordinates (0.15,0.3), (0.3,0.7), and (0.7,0.825) correspond to the cardiac segments *CS*_1_ = 0.15 - 0.3, *CS*_2_ = 0.3 - 0.7, and *CS*_3_ = 0.7 - 0.825, respectively. Figure 10(b) and (c) show one of the original image slices and the corresponding probability mapping (texture feature) for one of the high risk patients. The probability maps, which do not have 0 - 1 probability range, was extended using a sigmoid function for easy comparability. Figure 10(d–f) shows the cardiac segments corresponding to *CS*_1_, *CS*_2_ and *CS*_3_ for the image slice shown in Figure 10(b).

**Figure 11:**
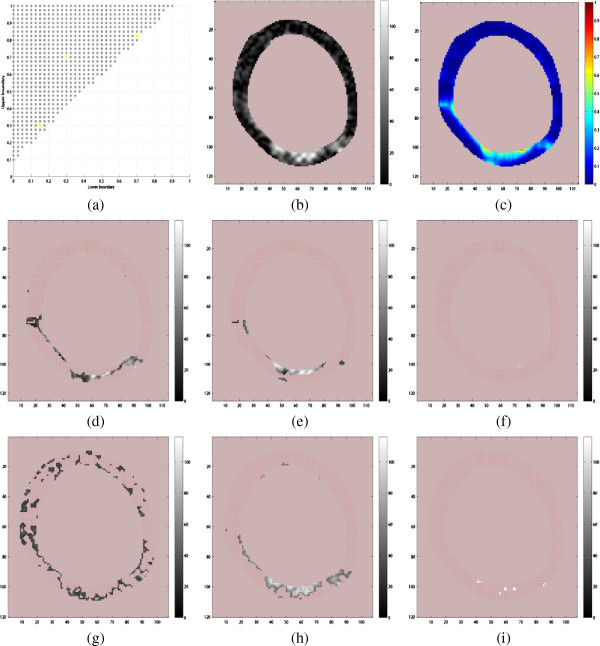
**Similar plots and cardiac segments as in Figure **10** for a patient from the group of patients with low risk of getting arrhythmia. (a)** The coordinates (*L*,*U*) shows all cardiac segments (CS) corresponding to the intervals *L* - *U*. **(b)** An image slice from a patient from the group with low risk of arrhythmia, **(c)** The corresponding probability map, and **(d–f)** the cardiac segments corresponding to the yellow (*L*,*U*) coordinates *CS*_1_, *CS*_2_, and *CS*_3_. Subplots **(g–i)** shows the corresponding cardiac segments derived without probability mapping.

Preliminary experiments indicate that corresponding segments determined without using the probability mappings depends on the signal intensity values and seems to lack the ability to define contiguous areas in the same way as the probability mapping for some of the cardiac segments. For example, the cardiac segment determined directly from thresholding relative to the maximum 3D scar signal intensity is shown in Figure 10(g–i) for boundaries corresponding to *CS*_1_*CS*_2_ and *CS*_3_, respectively. These plots are comparable to the corresponding cardiac segments based on the probability mapping in Figure 10(d–f), and show less contiguous cardiac segment, especially *CS*_1_. Figure 11 shows similar plots of the cardiac segments corresponding to *CS*_1_, *CS*_2_ and *CS*_3_ for one of the low risk patients.

**Figure 12:**
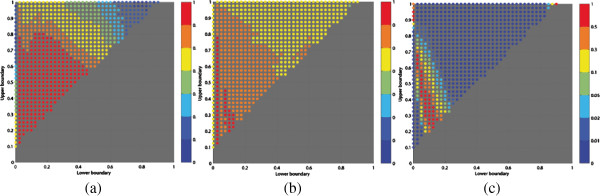
**Plot of (*L*,*U*) coordinates showing all cardiac segments (CS) corresponding to the intervals *L* - *U* comparing the relative sizes of the cardiac segments for two patient groups. **The color of the (*L*,*U*) coordinates indicates the p-value resulting from using the Mann-Whithney statistical test to determine if the relative sizes of the cardiac segments are different in the two patient groups. Subplots **(a)** and **(b)** corresponds to the tests of cardiac segments derived with probability mapping of texture and DC features, respectively. Subplot **(c)** shows the tests of cardiac segments derived directly from the original image.

For each candidate cardiac segment, its relative size (accumulated for all slices) to the total myocardium was calculated. The computed relative sizes for high and low risk patient groups were compared using a Mann-Whithney test at significance level 0.05. The LU-plots in Figure 12 show the p-values for the test of all the candidate cardiac segments derived using the texture based (a) and DC based (b) probability map and those derived without the use of probability mapping (c). The blue colored dots indicate cardiac segments for which the high and low risk groups showed significant differences of the relative size.

Interpreting the comparison results for the texture feature probability map based cardiac segments in Figure 12 (a), the significant CS with *L* = 0 and *U* > 0.5 correspond to the cardiac segment containing all of the healthy myocardium and gradually including the scar area. More interesting is the localization in the upper right corner which makes more specific localization in the myocardial tissue in the regions close to the scar. Note that *CS*_3_ is identified as significant while *CS*_1_ and *CS*_2_ are not. Figure 12 (b) shows the LU-plot from the probability maps of DC feature and, the plot shows that cardiac segments does not give any localization. This is due to the narrow transition between high and low probabilities of the DC based probability maps. The DC probability maps will predominantly have extreme values close to 0 or 1 and few values in between 0–1. This provides little information to explore. These plots (Figure 12 (a) and Figure 12 (b)) of cardiac segments clearly shows that the probability maps obtained from the DC features reflect the way cardiologist perceive the scarred myocardium in CMR images and, the probability maps from texture features gives information about the characteristics of myocardial tissue and might emphasize information that is not easy for inspection with a human eye in the original CMR image. The difference between the narrow and broad transitions for the DC- and texture-based probability mappings might explain this. As the narrow transition gives little information besides the crisp core/scar discrimination corresponding to the cardiologist’s delineation based on the visual information. Thus, most of the candidate cardiac segments will be empty as there are virtually no intermediate probability values. This is not the case for the texture based probability mappings, which discloses the information present in this intermediate probability range.

In comparison the results for the cardiac segments derived directly without using the probability plots (Figure 12 (c)), the localization seems vaguer, not specifically identifying any coherent region in the myocardium. It is our belief that probability mapping might be used to identify cardiac segments that might be used in future studies.

### The discriminatory power of the DC and texture features

**Figure 13:**
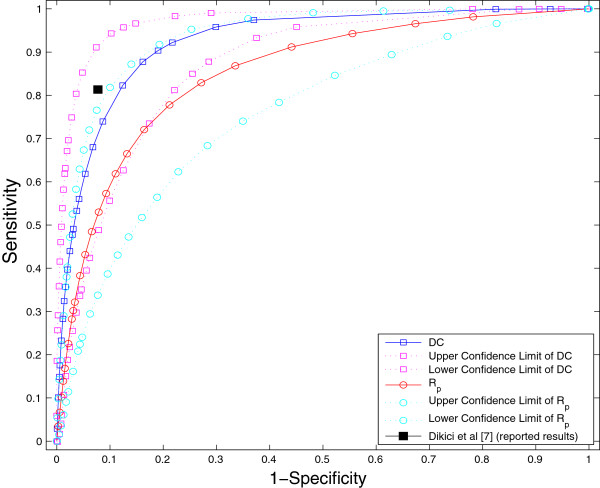
**The average ROC Curves with 95% confidence intervals for the DC and texture features on all the CMR slices of the18 test patients with high risk arrhythmia. **The ROC curves are compared to the results reported by Dikici *(et al.)*[7]. Area under the average ROC curves for the DC and texture feature are 0.9052 and 0.8428, respectively.

The probability maps calculated for the two features, DC and textural feature, give different information. The discriminatory power of the two features were explored by comparing to the manual segmentation of scar and healthy myocardium to obtain Receiver Operating Characteristic (ROC) curves. The ROC curves calculated for the DC and texture features on the CMR images of the 18 test patients with high risk of getting arrhythmia are shown in Figure 13. The area under the average ROC curves is 0.9052 and 0.8428 for the DC and texture features, respectively. From ROC curves, it is observed that the discriminative power of the DC feature is comparatively higher than that of the texture features. The sensitivity, and specificity reported in [7] are plotted as a single point in Figure 13. This reported result is *not* calculated on our CMR data. This single point lies below within 95% confidence interval of DC feature. This reported segmentation result performed well compared to our average DC and texture ROC curves as Dikici *et al.*[7] does not include all the CMRI slices of a patient for training and testing where as in our work we include all the CMRI slices i.e. the scar volume.

## Conclusion

In this paper, a new technique has been proposed for enhanced visualization of scarred myocardium in LG enhanced CMR images using probability mapping. Using Bayes rule and ML estimation, we obtained the probability maps of scarred myocardium using DC and texture features from each pixel in the myocardium. The probability values obtained from the DC features can be used for determining the scar size, and they reflect the way cardiologist perceive the scarred myocardium in CMR images. The probability maps from texture features gives information about the characteristics of myocardial tissue and emphasize information that is not easy for inspection with a human eye in the original CMR image. Using textural features in the probability mapping provide us with a tool for defining cardiac segments as well as give us features that can be used to distinguish between different groups of patients (patients high and low risk of getting arrhythmia). Our belief that the probability maps calculated by the texture feature captures the heterogeneous nature of scarred myocardium is justified with the example of the cardiac segments experiment. The two features tested in this paper gave valuable, but different, information. In future work, benefit of combining these two features as well as other features will be explored.

Intellectual Property Rights Patent pending. The Patent Application Number is 1121307.1. All Intellectual Property Rights are reserved by the University of Stavanger and Stavanger University Hospital. Prekubator TTO is responsible for IP management and commercialization.

## Appendix

### Recursive least squares dictionary learning algorithm

The dictionary learning problem can be solved in a two steps algorithm. Step 1) Find sparse coefficient matrix, *W*, using vector selection algorithms, keeping dictionary, *D*, fixed. Step 2) Keeping *W* fixed, find *D*. The dictionary update step depends on the dictionary learning method we choose to use. *D* and *C* are initialized randomly.

In this paper, an on-line dictionary learning algorithm, Recursive Least Squares Dictionary Learning Algorithm (RLS-DLA) presented in [17] is used for dictionary learning and ORMP vector selection algorithm presented in [16] is used for sparse representation. The RLS-DLA updates the dictionary with the arrival of each new training vector. In deriving updating rules for RLS-DLA, *X*_*t*_ = [*x*_1_,*x*_2_,…,*x*_*t*_] of size *S* × *t*, *W*_*t*_ = [*w*_1_,*w*_2_,…,*w*_*t*_] of size *K* × *t* and $C_{t}={\left(W_{t}W_{t}^{T}\right)}^{-1}$ are defined for the ’time step’ *t*. At each time step the dictionary *D*_*t*-1_ and the *C* matrix are updated so that they obey the least squares solution $D_{t}=(X_{t}W_{t}^{T}){\left(W_{t}W_{t}^{T}\right)}^{-1}$. The matrix inversion lemma (Woodbury matrix identity) is applied on *C*_*t*_ to get the following update rules:

(8) $$C_{t}\;=\;C_{t-1}-\mathrm{\alpha u}u^{T},$$

(9) $$D_{t}\;=\;D_{t-1}+\alpha r_{t}u^{T},$$

where *u* = *C*_*t*-1_*w*_*t*_ and $\alpha =1/(1+w_{t}^{T}u)$, *r*_*t*_ = *x*_*t*_ - *D*_*t*-1_*w*_*t*_ is the approximation error.

With the inclusion of an adaptive forgetting factor *λ*_*t*_ in RLS-DLA, the update equation 8 is changed to:

(10) $$C_{t}\;=\;(\lambda_{t}^{-1}C_{t-1})-\mathrm{\alpha u}u^{T},$$

where $u=(\lambda_{t}^{-1}C_{t-1})w_{t}$. The remaining equations remain the same. Due to introduction of the forgetting factor, RLS-DLA has good converging properties and is less dependent on the initial dictionary.

## Abbreviations

CMR: Cardiac magnetic resonance; MI: Myocardial infarction; LG: Late gadolinium; ICD: Implantable cardioverter defibrillator; SVM: Support vector machine; PDFs: Probability density functions; ML: Maximum likelihood; DC: Direct current; ORMP: Order recursive matching pursuit; RLS-DLA: Recursive least squares dictionary learning algorithm; NP-hard: Non-deterministic polynomial-time hard; FTCM: Frame texture classification method; VT: Ventricular tachycardia; DICOM: Digital imaging and communications in medicine; ROC: Receiver operating characteristic.

## Competing interests

The authors declare that they have no competing interests.

## Authors’ contributions

LPK carried out the feature extraction (DC and texture), probability mapping experiments and drafted the manuscript. KE and KS helped with the dictionary learning and sparse representation. TE participated in probability mapping and designed and performed the cardiac segment experiments. FM collaborated in finding the cardiac segments with out using the probability mapping. SØ and LW were responsible for the clinical studies including patient recruitment and acquiring of CMR images. They performed the segmentation of the healthy and scarred myocardium and contributed to the drafting and revision of the clinical and CMR related parts of the manuscript. KE, TE and KS also helped in drafting the manuscript. All the authors read and approved the final manuscript.
